# Pre-Loading of Cells via Vapor Sublimation and the Deposition Polymerization Process with a 3D Porous Scaffold for Cell Cultures

**DOI:** 10.1021/acsbiomaterials.5c00439

**Published:** 2025-07-10

**Authors:** Chung-Ju Chen, Chin-Yun Lee, Mei-Yu Chen, Ying-Hsuan Shi, Yu-Chih Chiang, Chen-Chi Wu, Hsien-Yeh Chen

**Affiliations:** † Department of Chemical Engineering, 33561 National Taiwan University, Taipei 10617, Taiwan; ‡ School of Dentistry, Graduate Institute of Clinical Dentistry, 33561 National Taiwan University, Taipei 10048, Taiwan; § Department of Otolaryngology, 38006 National Taiwan University Hospital, Taipei 10018, Taiwan; ∥ Molecular Imaging Center, 33561 National Taiwan University, Taipei 10617, Taiwan

**Keywords:** preload cell, scaffold, poly-*p*-xylylene, vapor sublimation and deposition, cell culture

## Abstract

In this study, we fabricate a three-dimensional (3D) porous poly*-p-*xylylene scaffold via a preloading technique and tailor it for cell culture. The fabrication process utilizes vapor sublimation and deposition polymerization, which exploits an ice template for sublimation and subsequent deposition of poly*-p-*xylylene under lower pressure and room temperature conditions. During this process, living cells are incorporated within a protective oil-in-water emulsion system, which facilitates high cell viability, and this construction forms a poly*-p-*xylylene scaffold with multiscale pores in the scaffold architecture that can be maintained for a tested time frame of 21 days in the current study. This reported fabrication method addresses inherent limitations of traditional methods, such as restricted biocompatibility, the need for modification procedures to achieve adequate porosity, and postseeding/loading of cells. By facilitating precise control over both micro- and nanostructures, the approach simultaneously preloads and accommodates multiple cell types and/or the necessary bioactive factors in the water solution and becomes an ice template. Finally, a single vapor phase fabrication step can lead to the construction of devised multifunctional scaffolds. The resulting scaffolds exhibit high porosity, featuring interconnected pores for cell migration and nutrient diffusion. Furthermore, controlled nanoroughness and microporosity promote cell attachment and enhance cell–cell and cell–matrix interactions, which are critical for tissue integration. Various types of cell cultures alongside diverse lineages of differentiations, including adipogenic, osteogenic, and neurogenic lineages, were examined in this study. Finally, the creation of anisotropic directional scaffolds that mimic native tissue architecture and promote cell attachment is particularly relevant for applications such as dental tissue regeneration and vascularization. Overall, the presented methodology represents a significant advancement in scaffold fabrication technology with considerable potential for versatility in regenerative medicine and complex tissue regeneration.

## Graphical abstract

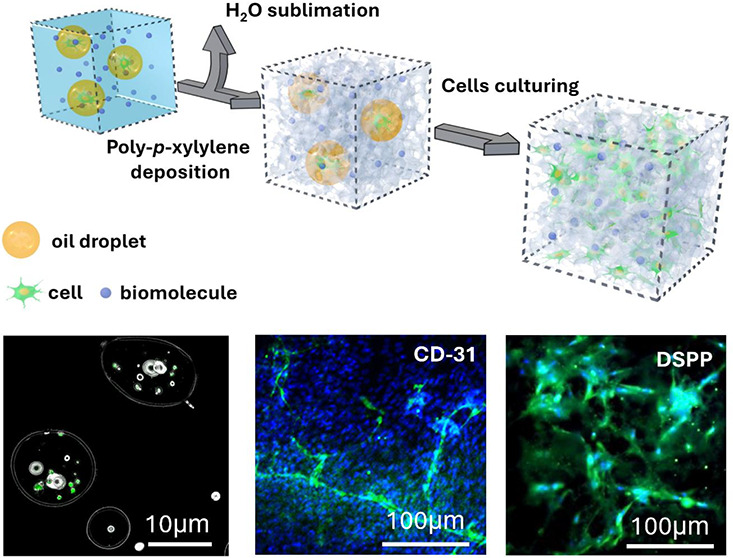

## Introduction

The development of advanced scaffolding materials represents one of the most transformative areas in the field of biomaterials and tissue engineering. In recent years, the integration of fabrication techniques such as vapor sublimation and deposition polymerization has introduced a paradigm shift in scaffold design, offering unprecedented control over micro- and nanostructures. Historically, traditional methods have been limited by their limited biocompatibility, insufficient porosity, and poor integration with host tissues. Additionally, the physicochemical properties of polymer scaffolds can influence their behavior, elucidating the complex interplay between scaffold topography and cellular interconnection.^1–3^ The development of advanced biomaterial scaffolds represents a critical frontier in addressing these limitations. Other fabrication techniques, such as electrospinning, 3D printing, freeze-drying, and salt leaching, while valuable, often produce structures with limited control over spatial organization, mechanical strength and biological incorporation simultaneously. Only approximately 30% of traditionally fabricated scaffolds achieve sufficient mechanical stability while maintaining adequate porosity for cellular infiltration.^4,5^ This fundamental limitation has driven the exploration of novel fabrication approaches that can better replicate the complexity of natural tissue environments. Vapor deposition polymerization has emerged as a promising alternative for scaffold fabrication, offering unprecedented control over structural features across multiple length scales.

The integration of multiple cell types and bioactive factors within a single scaffold system represents another significant challenge in tissue engineering. Traditional approaches often require sequential processing steps that can compromise biological activity and spatial organization. The development of single-step fabrication methods that can simultaneously incorporate various biological components while maintaining their functionality has become increasingly important. Previous studies have shown that maintaining growth factor bioactivity during scaffold fabrication can enhance tissue regeneration outcomes by up to 40%.^6,7^ Modular approaches in tissue engineering have gained particular attention because of their ability to create scaffolds with distinct functional domains. These systems demonstrate remarkable versatility in accommodating diverse cellular populations while maintaining precise control over the spatial organization of bioactive components.^8–10^ Research has demonstrated that, compared with single-factor systems, scaffolds incorporating multiple bioactive factors can enhance tissue regeneration efficiency by up to 60%.^11–14^ In addition, studies have established that scaffolds require specific pore size distributions to support different cellular functions, where pore sizes between 100–500 μm optimize cell migration and nutrient diffusion, whereas smaller pores enhance cell–cell interactions and cell adhesion.^15,16^ The development of scaffolds capable of supporting multiple tissue types simultaneously represents a frontier in tissue engineering research. Recent studies have shown that multitissue scaffolds can enhance functional integration, representing a significant advancement in complex tissue engineering.^17,18^ Scaffolds designed for adipogenesis, osteogenesis, odontogenesis, and neurogenesis can enhance the maturation, proliferation and differentiation of adipocytes, bone cells, dental stem cells and neuronal progenitor cells, which are crucial for reconstructing tissue.^19–22^ However, traditional scaffolds often fail to promote adequate blood vessel formation, limiting the size and complexity of engineered tissues.^23,24^ Various studies have shown that incorporating specific architectural features can greatly enhance vascularization compared with conventional designs.^25,26^

For 3D porous poly*-p-*xylylene scaffold fabrication, the concept of vapor sublimation and deposition polymerization is introduced as an innovative solution that combines the benefits of chemical vapor deposition with a controlled sublimation process.^27–30^ This method enables the formation of a 3D structure that is both porous and mechanically robust. The material chosen for scaffold fabrication—parylene, a comparable biocompatible polymer—provides the necessary chemical stability and resistance to degradation while simultaneously offering a surface that is conducive to the adsorption of growth factors and subsequent cell adhesion.^29,31^ Additionally, while homogeneous porous structures are commonly used in cell culture and tissue engineering applications, techniques such as patterning and 3D printing can facilitate multiple cell coculturing. However, the potential of anisotropic cell culture remains unrevealed.^32–35^

Anisotropy—or directional structuring—within a scaffold, i.e., the directional scaffold design, was further explored in this study, which elaborates on mimicking the organized structure of native tissues. This consideration is especially critical in dental tissues, where hierarchical organization is fundamental to achieving both mechanical resilience and proper physiological function.^36–38^ Directional cues are essential not only for guiding cell migration but also for engendering the correct spatial distribution of mechanical stresses across regenerating tissue. This aspect of scaffold design has been underexplored in numerous earlier studies, hence the novelty and potential impact of the present work. These structural attributes are critical for ensuring that newly formed tissue interfaces harmoniously with the surrounding biological environment, thereby minimizing complications such as mismatched mechanical properties or immune rejection.^9,39,40^ The originality of this study lies in the incorporation of bioreactive components and living cells within a single-step fabrication process, as well as the biomimetic anisotropic structures that mimic the organization of native tissues. These innovations provide valuable cues for the application of 3D poly-*p*-xylylene, particularly in tissue engineering and integration.

In this study, a 3D porous poly*-p-*xylylene scaffold with vapor deposition polymerization combined with homogeneous organization of bioactive factors and living cells was used to support various types of cell culture and to enhance cell differentiation. These techniques address several key limitations of conventional fabrication methods and the differentiability of scaffolds while offering new possibilities for complex tissue engineering applications. First, single-step fabrication with preloaded cells and bioactive factors preserves the bioactivity of incorporated factors and overcomes the challenges in multistep fabrication methods. Moreover, the preloading of cells within the scaffold using a protective oil-in-water emulsion system significantly enhances cell viability and distribution, overcoming the challenges associated with postseeding methods, which often result in uneven cell coverage and reduced survival rates. Second, high porosity (exceeding 90%) and precisely controlled microstructures, including controlled nanoroughness and microporosity, surpass traditional methods resulting in lower porosity (40–60%) structures and facilitate efficient nutrient transport, cell attachment, cellular behaviors and cell–scaffold interactions, addressing limitations in previous scaffold designs that hinder cell viability and tissue integration. Third, this approach allows for coculture and multicellular environments. Our fabrication method supports the incorporation of multiple cell types and bioactive factors within a single scaffold, enabling coculture systems with clear compartmentalization of biological activity. This overcomes the limitations of traditional methods that often struggle with integrating different cell types and maintaining their spatial organization, thereby fostering tissue-specific behavior and facilitating complex tissue engineering applications. Fourth, anisotropic design for guided cell growth, where directional structuring (anisotropy) mimics the organized structure of native tissues, is particularly relevant in dental tissue regeneration. This guides cell alignment and functional integration, addressing the limitations of previous scaffolds that often lack such directional cues, which are crucial for achieving both mechanical resilience and proper physiological function. Finally, the use of parylene as a scaffold material ensures superior biocompatibility and chemical stability, eliminating the cytotoxic residues associated with solvent-based methods and increasing the suitability of the scaffold for advanced tissue engineering applications.

## Results and Discussion

### Fabrication of 3D Porous Poly*-p-*Xylylene Scaffolds

In the present study, vapor-phase construction methodology represents a critical progression in scaffold fabrication, allowing the deposition of structural and bioactive components in a single fabrication process. As illustrated in Figure 1a, the procedure begins with a finely tuned ice template that underpins controlled poly*-p-*xylylene deposition at rates of 0.1–0.5 nm/s. Following this deposition, a vapor sublimation process exploits the self-sacrificing template, generating a complex internal architecture while maintaining the organization of bioactive factors and living cells. This study expands on previous vapor-phase depositions of biomaterials, focusing on static or single-component materials,^41–44^ by enabling simultaneous structural and biochemical manipulation. The key technology of the scaffold fabrication method lies in incorporating living cells under otherwise challenging processing conditions by employing an oil-in-water emulsion system, overcoming prior limitations in vapor-deposited scaffolds, and preserving high cell viability during the low-pressure gas-phase reaction responsible for generating the 3D porous poly*-p-*xylylene scaffold. In this process, the oil phase envelops cells, offering partial isolation that allows them to survive deposition while concurrently forming the structural poly*-p-*xylylene scaffold. This approach leverages the nonpolar characteristics of both the oil and the parylene precursor to enable mutual diffusion on the scaffold surface, effectively “releasing” preloaded cells into the fabricated porous scaffolds. When the scaffold is moved into the culture medium, continuous media exchanges further sustain high viability, reducing acute toxicity and minimizing uncertainty in the subsequent cell culture steps. Unlike many benchmark bioscaffolds that often trigger significant inflammatory responses and require a protracted adaptation period, this engineered scaffold framework shortens the acclimatization phase by employing homogeneously controlled bioactive molecules, thereby expediting early cell–scaffold interactions and potentially accelerating tissue repair, suggesting shorter clinical recovery times in regenerative applications.^45–47^

**Figure 1:**
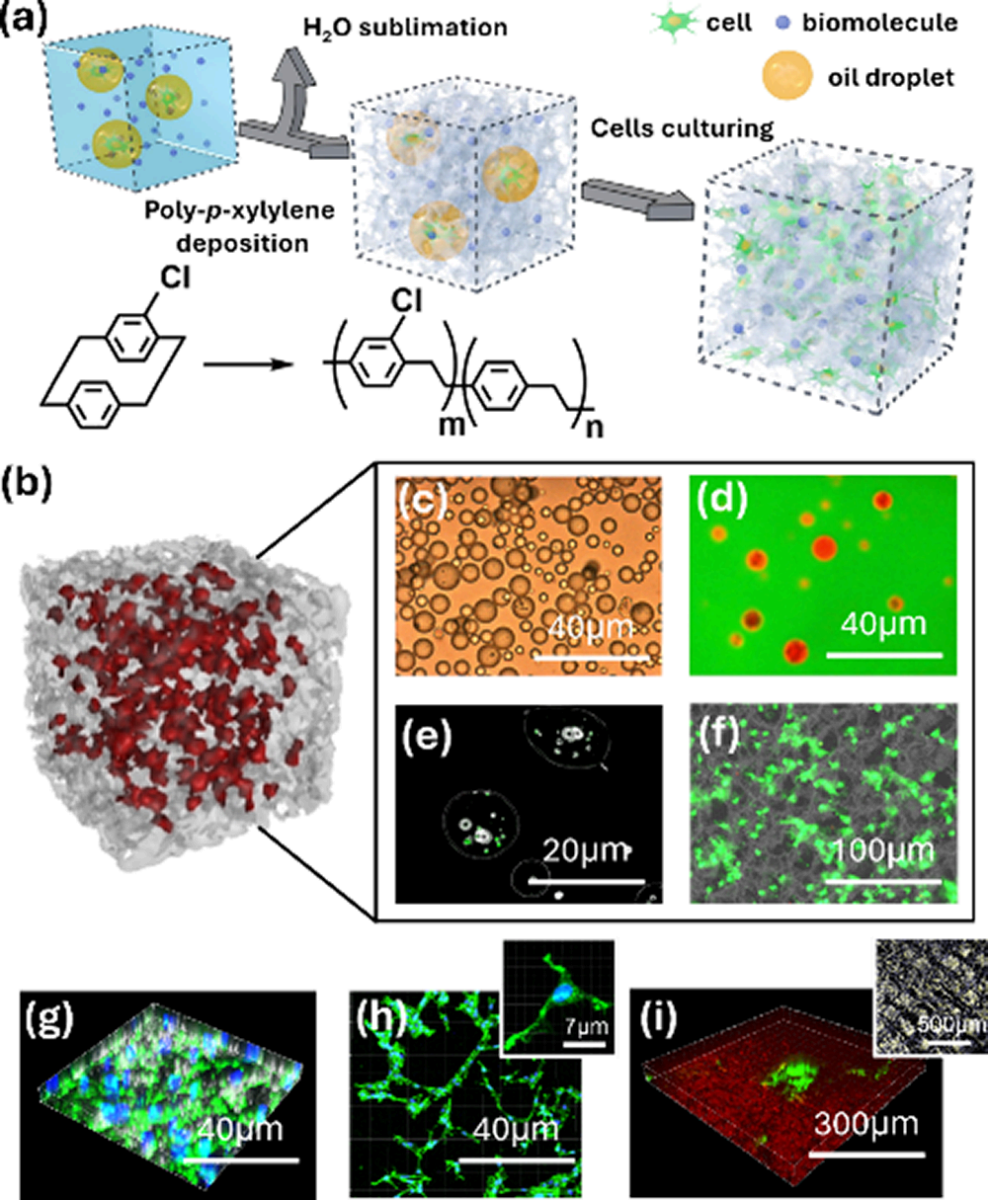
3D porous poly*-p-*xylylene scaffold fabrication process and biocompatibility. (a) Schematic of the controlled scaffold deposition/sublimation process of an ice template and cell viability in a 3D porous poly*-p-*xylylene scaffold. (b) Micro-CT reconstruction of the scaffold filled with MG-63 cells (red). (c) Bright-field microscopy image of oil droplets and (d) fluorescence microscopy image of oil red O in the oil phase and the FITC-labeled water phase in an oil-in-water system. (e) Phase-contrast fluorescence microscopy image of living cells (MG-63) in an oil-in-water system and (f) high-resolution SEM image of the MG-63 integrated LIVE/DEAD fluorescence signal (green) on the scaffold surface after 21 days of culture. (g) 3D and (h) 2D confocal microscopy images of MG-63 cells after 21 days of culture, showing the distributions of the cell body (green) and cell nuclei (blue) within the biocompatible architecture of the scaffold stained with LIVE & DEAD and DAPI, respectively. (i) High-resolution 3D confocal microscopy image of microlevel cocultures control over MG-63 (red) and HUVECs (green), as evidenced by a small SEM image nearby.

The stable oil-in-water system under optical microscopy and fluorescence microscopy, as part of preliminary experiments, confirmed the successful demarcation of hydrophobic and hydrophilic domains, as shown in Figure 1c,d. Such dual-phase systems have found utility in drug delivery scenarios;^48–50^ however, our incorporation of living cells into these phases highlights a substantial step forward for tissue engineering. In accordance with the different phases underpinning the scaffold’s ability to accommodate multiple cell types,^51,52^ previous studies also revealed that 82.5 ± 3.2% of cells persisted in a metabolically active state for at least 21 days,^35,41,53^ a result notably higher than that of conventional encapsulation methods (60–70%).^54–56^ This underscores the importance of a benign vapor-phase process in preserving cellular and molecular integrity—a critical necessity in advanced tissue engineering contexts. On the other hands, prior studies have shown that postseeding often leads to uneven cell distribution, underscoring the need for preloaded cell integration and proper 3D assessment.^57,58^ To highlight these benefits, we used 2D TCPS as a control to demonstrate the overall biocompatibility and enhanced differentiation of our 3D scaffold design.

For the realization of the deposition process of parylene preloaded with cells, the cryoprotectant medium included living cells such as ADSCs, DPSCs, PC12, HUVECs, and MG-63, along with the growth factors BMP-2, FGF-2, VEGF, and PRP, which were selected and solidified with liquid nitrogen at −196 °C, which helped to create an ice template and prevent any mixing of the components. The cells and biomolecules were centrifuged and suspended in an oil-in-water system to preserve distinct phases for rapid imaging using phase-contrast microscopy, as illustrated in Figure 1e. This modulation involved creating an ice template from a solution that had specific solute compositions, carefully avoiding the use of sacrificial dopants that were common in previous studies.^59,60^ Immediately after fabrication and removal from the deposition chamber, the experimental samples were immersed in culture medium to maintain cell viability. Subsequent analyses confirmed the survival of localized cells within the oil-in-water system through LIVE/DEAD fluorescence staining and SEM imaging, which revealed cellular integration within the scaffold of MG-63 cells after 14 days of culture, as depicted in Figure 1f. Furthermore, the SEM background illustrates the distribution of cells on the scaffold surface and their passage through the inner pores and fiber structure of the scaffold, as shown in Figure 1f. This observation demonstrates the consistency of cell migration and proliferation within the scaffold structure, highlighting the intimate relationship between cells and the engineered scaffold. This result is consistent with previous studies using the same fabrication method, as it demonstrates that over 80% of the cells successfully survived the cell preloading and fabrication process, as confirmed by ImageJ analysis relative to the TCPS control.^61^ Moreover, these findings align with previous research, which underscores the significant impact of growth factors on cellular behavior within scaffolds. Specifically, studies have demonstrated that FGF-enriched environments not only boost cell viability and proliferation but also play a crucial role in promoting differentiation.^41^ Additionally, the freezing process did not significantly affect the viability and differentiation potential of the cells. As demonstrated in our previous study by Wu et al., which employed the same fabrication process to create the cell-alive scaffold, 96.1% of the cells remained alive immediately after freezing and 80.8% after the fabrication in the final scaffold constructs.^41^ Furthermore, as shown in Figure 1f, over 80% of the cells maintained viability within the scaffold. Other research also confirmed no significant differences in viability or recovery rates for undifferentiated cells subjected to the freezing process.^62^

Comparable findings have been reported in advanced tissue engineering studies, emphasizing the importance of maintaining bioactive compositions during fabrication.^63–65^ Micro-CT analyses, as shown in Figure 1b, revealed a highly porous structure with interconnected channel systems. MG-63 cells are visualized in red against a translucent background, illustrating the nature of the cell distribution within the scaffold and indicating high biocompatibility and potential for cellular regulation within the scaffold. 2D and 3D confocal microscopy images, as shown in Figure 1g,h, further revealed complex cellular arrangements, suggesting a supportive microenvironment conducive to cell–cell and cell–scaffold interactions.

Previous studies have demonstrated that heterotypic cellular interactions underpin many fundamental processes in tissue formation,^66–68^ and our approach capitalizes on this principle by enabling coculture within a single scaffold. In addition, research on cell adhesion and cell–cell interactions indicates that geometric patterning enables precise control over directional growth and cell adhesion, facilitating the shortest path from edge to edge.^69–72^ Meanwhile, spatial growth factors can modulate cell–cell interactions by providing protein signaling connections within the extracellular matrix, thereby influencing the resulting patterns.^73–75^

Here, the square patterns observed by 3D fluorescence confocal microscopy in Figure 1i reflect the reliability of the fabrication process in sustaining patterned cell growth. The distribution of red and green fluorescent markers clearly indicates compartmentalization between MG-63 cells and HUVECs, which is vital for mimicking native tissue architecture and complex tissue engineering. This cell combination was intentionally selected based on histological evidence indicating that osteoblasts and osteoprogenitor cells are typically located adjacent to vascular endothelial cells in native human bone tissue, as demonstrated in previous studies.^76,77^ The spatial arrangement is vital for mimicking native tissue architecture and function. These results underscore how precise geometric control within the scaffold can promote distinct cell distributions, folding into an interconnected microenvironment that fosters tissue-specific behavior. This technique within scaffold fabrication has drawn substantial attention to vapor-phase methodologies, particularly for tissue engineering applications, which integrate multiple bioactive elements and diverse cell types within 3D scaffolds, thereby offering superior control over scaffold architecture and biological functionality.

### Characterizations of 3D Porous Poly*-p-*Xylylene Scaffolds

After the sublimation and deposition fabrication process, the 3D porous poly*-p-*xylylene scaffold was characterized in detail. This technique can create highly organized architectures while preserving biological activity, achieving porosity levels over 90% and improving structural integrity. This enhanced porous structure was validated by mercury porosimetry and micro-CT analyses, as shown in Figure 2b–d, respectively. The main pore size distributions, ranging from 1–150 μm, as shown in Figure 2c, are critical for cell migration, vascularization, and interconnectivity. These morphological features are known to facilitate efficient nutrient flow and biochemical signaling, mirroring the design principles observed in other high-porosity scaffolds.^15,78,79^ Notably, the interconnectivity index (approximately 85%) and the reduced tortuosity factor (approximately 1.3) suggest robust pathways for cellular infiltration across the scaffold. These findings corroborate prior studies, which reported improved cell infiltration and tissue integration in scaffolds featuring hierarchical pore networks.^80,81^ The SEM image in Figure 2e aligns with the micro-CT reconstructions shown in Figure 2d, confirming the existence of macropores and highlighting the hierarchical organization supporting cell adhesion. The macroscopic and reconstructed view in Figure 2a, which the scaffolds are supported by cotton fibers, reveals a controlled scaffold design of cylindrical examples and possesses lightweight property. The structural symmetry index of 0.85 is consistent with a highly porous morphology, suggesting future applications for load-bearing tissue constructs or specialized organoid culture systems.

**Figure 2:**
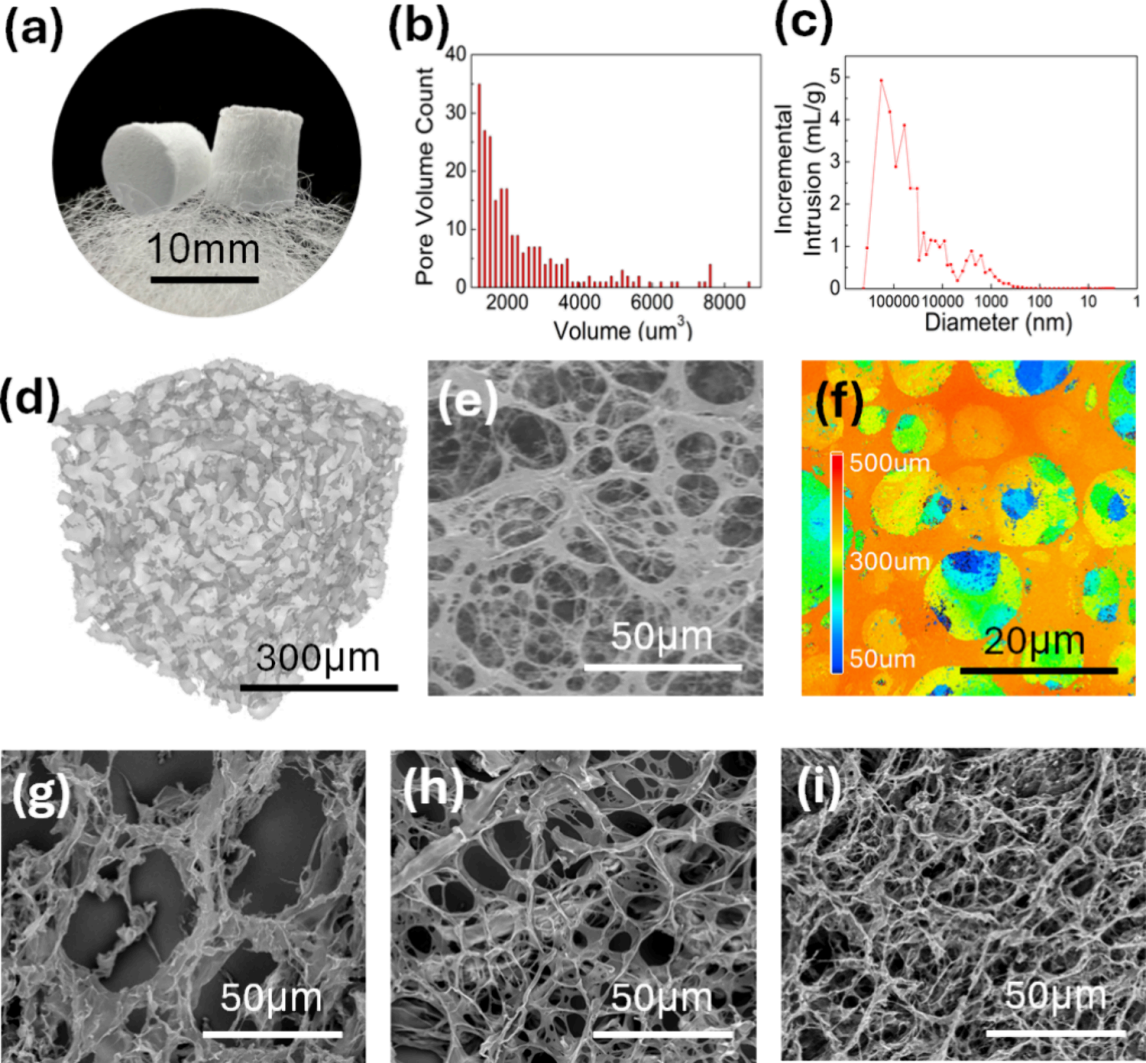
Characterization and quantitative analysis of the pore size of the 3D porous poly*-p-*xylylene scaffold in terms of (a) macroscopic view, (b) pore volume distribution and (c) pore size distribution. (d) Micro-CT image shows an overview and detailed interconnected structure with surface topography by (e) SEM analyses and (f) compositional mapping via 3D-laser confocal microscopy, which visualizes distinct material regions with varying depths. The higher regions marked with warm colors and the lower regions with cooler colors. Multiscale image of the 3D porous poly-*p*-xylylene scaffold from the larger structural level to the smaller structural level, demonstrating different structural examples: (g) large pores (pore sizes of 50 μm), (h) medium pores (pore sizes of 15 μm), and (i) small pores (pore sizes of 5 μm).

In addition, nanoroughness and microporosity on scaffold walls play critical roles in mediating protein adsorption and cellular communication. The SEM image in Figure 2e shows an overview of the scaffold surface with a fiber-like texture, which provides favorable topographical cues for future tissue engineering applications.^82,83^ These findings align with broader evidence that surface roughness at the micro- and nanoscales can significantly increase cell attachment, proliferation, and differentiation.^84,85^ Compositional mapping, measured by 3D-laser confocal microscopy and depicted in Figure 2f, illustrates the scaffold surface topography. The different colors correspond to varying height levels, where warmer colors indicate higher regions and cooler colors indicate lower regions. This mapping also sheds light on the spatial distribution of material components at different depths. Such selective layering potentially enables the formation of specialized microdomains for cell populations or nutrients, supporting demands within the same construct. Additionally, this capability facilitates the creation of specialized microstructures within the scaffold, offering valuable potential for applications in specialized tissue engineering that require multiple cell types or varying mechanical properties. These design considerations, which merge advanced processing with functionality, are increasingly pivotal in tissue engineering strategies that integrate multiple cell types for complex tissue repair.

Further structural insights (Figure 2g–i) revealed discrete multiscale features resulting from carefully regulated deposition parameters. Notably, the correlation between architectural precision and cellular behavior underscores how controlled tortuosity and interconnectivity promote effective nutrient and waste exchange, which is a critical requirement in tissue constructs.^86–88^ The larger-scale structure, as shown in Figure 2g, has a pore size of approximately 50 μm, facilitating efficient nutrient transport. These characteristics make the scaffold particularly suitable for oxygen-dependent tissues such as lung and liver applications. In contrast, the smaller pore architecture shown in Figure 2i could enhance cell migration, proliferation, and the formation of the extracellular matrix while promoting cell–cell interactions.^89–91^ This configuration is particularly advantageous for tissues requiring strong surface adhesion and cell junction, such as skin and blood vessels. For further investigations, we selected scaffolds with the intermediate structural parameters shown in Figure 2h, aligning with emerging principles that advocate a balance between nutrient transport and cell adhesion.

In brief, these results validate an innovative strategy to achieve high porosity, enhanced interconnectivity, and preserved cellular viability within a 3D scaffold. Unlike conventional freeze-drying, this approach involves ice-templating to obtain precise pore geometries and surface textures. The comprehensive analysis shown in Figures 1 and 2 underscores how the scaffold geometry, pore architecture, and surface microscale nuances support cell culture and direct cell behavior and coculturing and further represents a future direction of advancement in tissue-specific regeneration fields.

### Guiding Cell Differentiation

In differentiation experiments, the 3D porous scaffold not only supports but also actively modulates multiple lineage commitments. Previous studies have shown that the geometry of the scaffold and the morphology of stem cells play a crucial role in modulating lineage-specific differentiation. This effect is influenced by factors such as cell adhesion and cell–cell interactions, which can be adjusted by geometry and mechanical properties of the scaffold.^92–94^ Additionally, the differentiation experiments employed a specific concentration of growth factors, as detailed in methods. With regular medium refreshment, this concentration stabilizes, finally enabling the scaffolds to enhance differentiation compared to the TCPS control, as the result shown in Figure 3.

**Figure 3:**
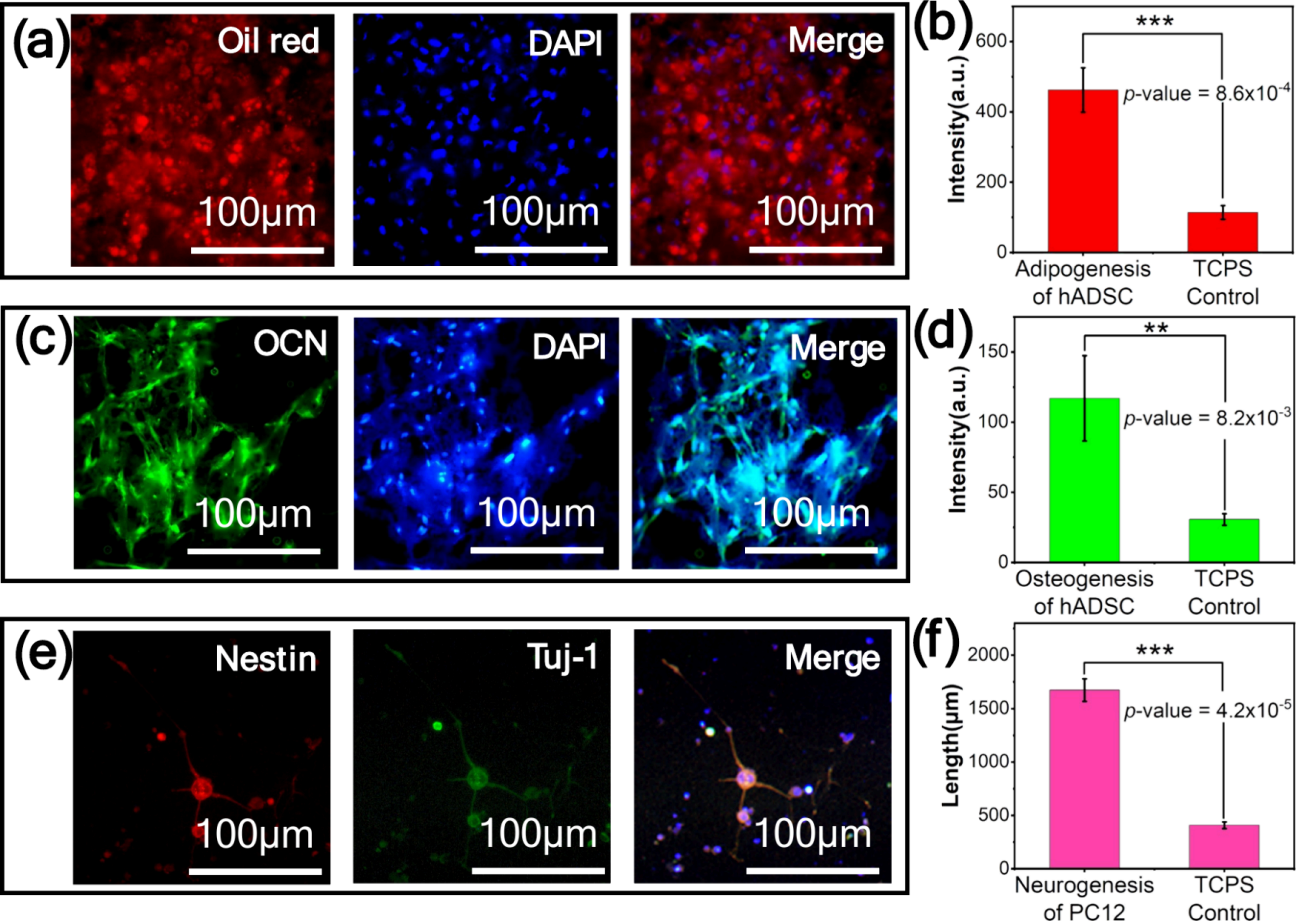
Cell differentiation within a 3D porous poly*-p-*xylylene scaffold. (a) Fluorescence confocal microscopy of the adipogenic differentiation of ADSCs stained with Oil Red O (red) and DAPI (blue) showing (b) 4.1-fold greater fluorescence signals than those in the TCPS control group after 21 days of culture. (c) OCN immunofluorescence of ADSC osteogenesis, stained with an OCN immunoprotein marker (green) and DAPI (blue), with a (d) 3.8-fold greater fluorescence signal than that of the TCPS control group after 21 days of culture. (e) Neural differentiation immunofluorescence image of PC12 cells stained with Nestin (red) and Tuj1 (green) protein markers with (f) neurite outgrowth 4.2-fold greater than that of the TCPS control group after 21 days of culture (*n* = 3, **: *p* value <0.01; ***: *p* value <0.001).

In adipogenic differentiation assays using ADSCs, Oil Red O staining confirmed a notable increase in lipid accumulation compared with that in the control group, as shown in Figure 3a, reflecting the capacity of the scaffold to influence cellular outcomes through microstructural cues and to organize the cell–cell interaction within the scaffold well. These observations resonate with the quantified analysis of Oil Red O (red) fluorescence signals, revealing that ADSC adipogenesis was 4.1-fold greater than that in the TCPS control group after 21 days of culture, as shown in Figure 3b, highlighting the subtle interplay between scaffold design and cellular protein expression. Furthermore, the Oil Red signal within the scaffold was expected to be eliminated based on the previous study by Chiu et al.,^95^ and research by Chang et al. supports the idea that the combination of growth factors and inducing factors with the scaffold can enhance cell differentiation.^96^ In addition, osteogenesis stimulated by the same scaffold system manifested as a 3.8-fold increase in OCN expression, as shown in Figure 3d, and fluorescence confocal images of OCN immunofluorescence (green) and DAPI (blue) of 21-day-cultured ADSCs further support the numerical results, as shown in Figure 3c. This observation corroborates studies that link well-structured frameworks to enhanced mineral deposition and bone-like tissue formation.^97–99^ The emergence of a robust OCN-positive architecture signals efficient recapitulation of native bone microstructures—a factor integral to long-term stability and function in engineered bone grafts. In parallel, neural lineage differentiation was explored with immunofluorescence images of PC12 cells stained with Nestin (red) and Tuj1 (green), which are signature protein markers of neural differentiation, as shown in Figure 3e, revealing extensive neurite outgrowth. The quantitative measurements revealed a 4.2-fold greater increase in neurogenic activity than that of the traditional scaffold controls, as shown in Figure 3f. The arrangement noted for these extending neurites aligns with previous demonstrations of topography-driven neural differentiation.^100,101^ This capacity to nurture both mesoderm-derived osteogenic/adipogenic lineages and ectoderm-derived neuronal cells stands in stark contrast to older biomaterial platforms, which typically offer restricted lineage potential.^102,103^ Furthermore, our recent research has demonstrated the utilization of advanced scaffold technologies to enhance the differentiation potential of stem cells. Specifically, the methods reported by Christy et al.^104^ exemplify substantial advancements in scaffold design, guiding differentiation of DPSCs into functional odontoblasts and Wu et al.^41^ demonstrated angiogenesis through CD31 expression in HUVECs and osteogenesis via type-I collagen (COL-I) expression in MC3T3-E1 cells. These previous studies have shown similar results of various cell types differentiation, which is consistent with our experimental data.

Here, our results indicate that 3D porous poly-*p*-xylylene scaffolds can effectively induce differentiation in various cell types, and the use of ADSCs in Figure 3a,c demonstrates that the modulation of biomolecules within the scaffold can direct the same population of stem cells toward different desired cell phenotypes. This finding suggests a significant contribution to the future of complex tissue engineering by potentially enabling more precise regional tuning and greater differentiability of stem cells compared with previous studies.^105–111^ In summary, these results underscore the versatile nature of our 3D porous scaffold in directing multiple lineages, such as adipogenic, osteogenic, and neurogenic lineages, through its microstructural cues and organized cell–scaffold interactions, revealing its potential for complex-tissue engineering applications.

### Structural Alignment Modification of Scaffolds

Moving beyond single-lineage applications, these experiments introduce an enhanced vapor deposition polymerization technique that combines aligned structures to fabricate 3D “pipelined” scaffolds. This enables an integrated environment in which the cell types proliferate in aligned tissue structures such as DPSCs and HUVECs. The structural coherence of these systems was established by micro-CT reconstructions, as shown in Figure 4a; optical microscopy, as shown in Figure 4b; and confocal mapping, as shown in Figure 4d. The parallel striation patterns reminiscent of those of native tissue align with prior reports that highlight the importance of directed topographical cues for orchestrating cell growth.^112,113^ Similarly, ongoing improvements in dentinal tubule engineering have informed our scaffold’s biomimetic design for dental applications.^104^ Subsequent cellular analyses, as shown in Figure 4c, revealed that DPSCs aligned along engineered channels, reinforcing their architectural role in driving odontoblast-like differentiation. The complementary EDS profiles shown in Figure 4e confirmed that the mineralized deposits were consistent with active dental pulp formation, echoing the findings of prior mineralization studies.^114–116^ Additional characterizations of the pipeline structures via micro-CT analysis and SEM (Figure 4f,g) confirmed their uniformity. The circular cross section in Figure 4f shows the homogeneous internal pipeline structure and tunnel distribution with vertical and horizontal demarcations, as shown nearby Figure 4f. This result indicates the presence of a 3D porous tubular architecture within the scaffold, which is essential for guiding odontoblast organization during dentin formation.

**Figure 4:**
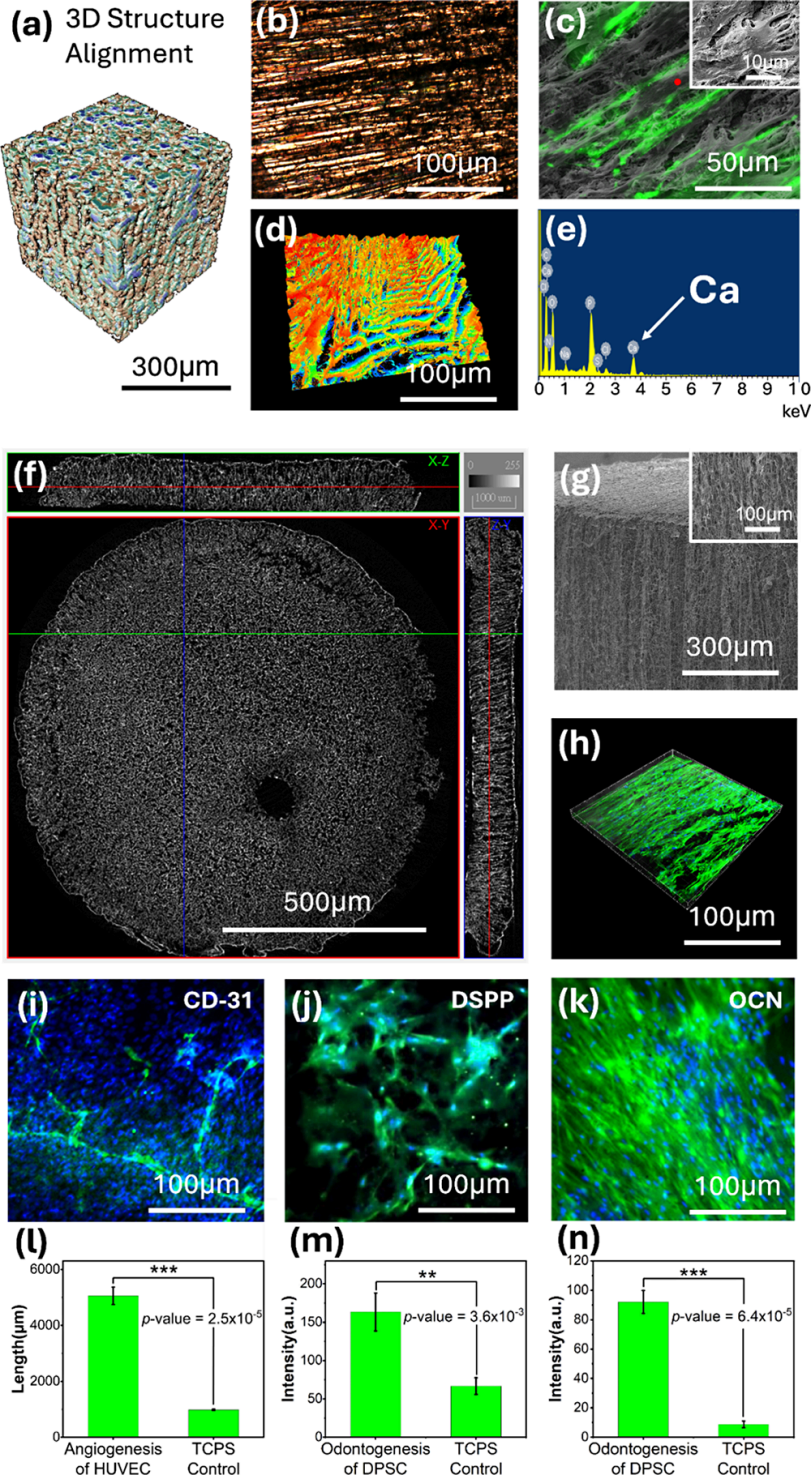
Characterizations of 3D-aligned poly*-p-*xylylene scaffold with cell culture and differentiation. (a) 3D micro-CT reconstruction model of the complex internal architecture within the scaffold, featuring an interconnected porous pipelined alignment. (b) Optical microscopy image of the scaffold surface with parallel striations, and (d) 3D laser confocal mapping image of the surface roughness and topology of the alignment scaffold, where the micrometer-scale depth is shown by false-color height variations. (b) Fluorescence microscopy and SEM images of DPSCs elongating through the scaffold topography and (e) EDS data of calcium mineralization and the basic material (parylene) of carbon and chlorine (f) micro-CT cross-sectional analysis of the internal pipeline structure over 1 mm and (g) macroscopic SEM view of the scaffold-aligned structure. (h) 3D laser confocal vertical biopsy images of DPSC proliferation within the scaffold stained with LIVE & DEAD (green) and DAPI (blue) after 21 days of culture. (i) Fluorescence microscopy of angiogenesis in HUVECs stained with the CD31 protein marker; (l) the CD31-positive signal was 5.2-fold greater than that in the TCPS control group after 21 days of culture. (j) Odontogenic immunofluorescence imaging of DPSCs stained with a DSPP antibody (green) and DAPI (blue), with (m) a 2.5-fold increase compared with that of the TCPS control group after 21 days of culture. (k) OCN immunofluorescence of DPSC odontogenesis, which was performed by staining with an OCN immunoprotein marker (green) and DAPI (blue) with (n) a 10-fold greater fluorescence signal than that in the TCPS control group after 21 days of culture (*n* = 3, **: *p* value <0.01; ***: *p* value <0.001).

These observed architectural features are similar to those of the natural dentin tubular structure,^117,118^ suggesting the potential for biomimetic tissue regeneration. Related research by Nur et al. likewise highlights the functional importance of cell-instructive substrates featuring precise topographies.^119^ The 3D confocal fluorescence image in Figure 4h shows a vertical biopsy of DPSCs in the scaffold. The green fluorescence labeling revealed that DPSCs elongated through the microchannel in the scaffold after 21 days of culture, and the observed cellular distribution patterns suggest that effective nutrients support cell proliferation, confirming the biocompatibility and flexibility of cell culture within the “pipelined” aligned structure.

Critically, the scaffold supported robust angiogenesis, as evidenced by extensive CD31-positive HUVEC networks displaying a 5.2-fold increase in vascular formation rates, as shown in Figure 4i,l. These findings address the longstanding challenge of vascularizing engineered tissues, thereby enabling efficient nutrient distribution in larger grafts. Comparative studies validating vascular network complexity parallel our observations and emphasize the importance of microenvironmental cues in endothelial network formation.^102,120^ Additionally, odontogenic differentiation, as demonstrated by the expression of dentin sialophosphoprotein (DSPP) and OCN, as shown in Figures 4j,k,m,n, was enhanced up to 2.5- and 10-fold relative to that of the control scaffolds, indicating robust dental tissue formation. Previous studies identified these markers as informative indicators of functional dental development, underscoring the success of the scaffold in recapitulating the natural sequence of tooth formation.^121–123^

Overall, the multifunctionality of our porous scaffold, which simultaneously supports neurogenesis, adipogenesis, osteogenesis, angiogenesis, and odontogenesis, opens new prospects for advanced regenerative medicine. Traditional approaches, often limited by single-lineage specialization, struggle to replicate the interplay of multiple tissue types and microenvironments. The enhanced vascularization observed herein could prove pivotal for scaling constructs to clinically relevant sizes—a longstanding bottleneck in the field. Similarly, the successful recapitulation of odontogenic processes contributes valuable insights into dental pulp regeneration, aligning with progressive strategies aimed at replacing or rebuilding compromised dental tissue. In this study, three interconnective properties, namely, precise microarchitectural control, multilineage differentiation potential, and high cell viability, exemplify the type of integrated scaffold technology that is foundational for complex tissue regeneration. These results integrated with tissue engineering frameworks provide foresight for next-generation biomaterial designs.

In summary, these results underscore the sophistication and versatility of our vapor–phase deposition technique, enabling the seamless fusion of structural intricacy with robust biological functionality. By combining novel fabrication parameters with a cell-protective environment, the results significantly broaden the potential of 3D scaffolds to diverse differentiation pathways in tandem. These findings further validate that multifaceted, biologically coherent scaffold designs are crucial for replicating native tissue complexity and driving successful regenerative outcomes. Future research will expand iterations of this technology of advanced biofabrication strategies for organ-level constructs and explore clinical applications that demand rapid and reliable tissue integration.

## Conclusions

A 3D porous poly*-p-*xylylene scaffold integrating living cells with high viability and biochemical modulation via the vapor–phase deposition technique was implemented. The scaffold exhibited high porosity (>90%), interconnectivity and biocompatibility, exceeding conventional methods and promoting cell adhesion, proliferation, and differentiation for multiple cell types, including ADSCs, DPSCs, MG-63 and HUVECs. Micro-CT and SEM analyses confirmed the intricate pore architectures of oil–water systems, including microscale topographies and multiscale features, facilitating efficient nutrient flow and robust cell–scaffold interactions for tissue integration. The scaffold preserved more than 80% of the viable cells at 21 days, which was greater than that of traditional encapsulation methods. This structure supports distinct cellular interactions and precise geometric patterning that promote lineage-specific differentiation, as evidenced by enhanced adipogenic, osteogenic, and neurogenic differentiation, and facilitates robust angiogenesis and odontogenic differentiation. Overall, these results validate the potential of the scaffold to facilitate cell–scaffold interactions, offering promising versatility in regenerative medicine and complex tissue engineering.

## Materials and Methods

### Materials

Dichloro*-p-*cyclophane (GALXYL C, Galentis, Italy) was purchased from La Chi Enterprise Co., Ltd. (Taiwan). Hydrochloric acid (36.5–38.0%, ACS reagent) was purchased from Honeywell Fluka (Charlotte, NC, U.S.A.). Sodium chloride (≥99.0%, ACS reagent) and sodium hydroxide (≥98%, reagent grade) were obtained from Sigma-Aldrich (St. Louis, MO, U.S.A.). Blue dye (Brilliant Blue FCF, Warner-Jenkinson Co., St. Louis, MO, U.S.A.) was purchased from Echo Chemical Co. (Taiwan). The polydimethylsiloxane (PDMS) used for mold fabrication was purchased from Dow Corning (Midland, MI, U.S.A.). Glyceryl trioleate was obtained from Sigma-Aldrich (St. Louis, MO, U.S.A.). Fibroblast growth factor-2 (FGF-2, 5 μg/mL) and Wnt-3a protein were purchased from R&D Systems (Minneapolis, MN, USA). Carboxymethyl cellulose (CMC) was purchased from Sigma-Aldrich (St. Louis, MO, U.S.A.). The cell culture media and supplements used included MEM-alpha, M199, Roswell Park Memorial Institute (RPMI-1640), and Dulbecco’s modified Eagle’s medium (DMEM), which were procured from Thermo Fisher Scientific (Waltham, MA, U.S.A.). Fetal bovine serum (FBS) and antibiotic-antimycotic mixtures were obtained from Biological Industries (Kibbutz Beit-Haemek, Israel). Dimethyl sulfoxide (DMSO), acetamide, propylene glycol, and polyethylene glycol (PEG, M.W. 8000) were purchased from Sigma-Aldrich (St. Louis, MO, U.S.A.). Additional supplements included dexamethasone, l-ascorbic acid, β-glycerol phosphate, insulin, 3-isobutyl-1-methylxanthine, all-trans retinoic acid, basic fibroblast growth factor (bFGF), epidermal growth factor (EGF), vascular endothelial growth factor (VEGF), heparin, and ECGS, all sourced from Sigma-Aldrich (USA).

Human adipose-derived stem cells (ADSCs) were obtained from the American Type Culture Collection (ATCC, USA) under catalog number PCS-500-011. The PC12 cell line, derived from rat adrenal pheochromocytoma cells, was also obtained from ATCC (CRL-1721). The MG-63 human osteosarcoma/osteoblast cell line was acquired from the Bioresource Collection and Research Center (BCRC, Taiwan) under catalog number 60279. Human umbilical vein endothelial cells (HUVECs) were obtained from HBCRC (H-UV001). Human dental pulp stem cells (DPSCs) were isolated from healthy third molars or premolars of patients aged 20 to 45 years, with explicit informed consent. The study was approved by the Institutional Review Board at National Taiwan University Hospital. The CellTracker Green CMFDA and PKH26 Red Fluorescent Cell Linker were purchased from Thermo Fisher Scientific (Waltham, MA, U.S.A.). The LIVE/DEAD Viability/Cytotoxicity Kit was also obtained from Thermo Fisher Scientific. For immunostaining, osteocalcin (OCN), β-III tubulin (Tuj-1), and cluster of differentiation 31 (CD31) antibodies were procured from Abcam (Cambridge, U.K.). The anti-Nestin antibody was obtained from Abcam (Cat. No. ab6142). Dentin sialophosphoprotein (DSPP) antibodies were purchased from Santa Cruz Biotechnology (Dallas, TX, U.S.A.). Oil Red O staining reagent was obtained from Sigma-Aldrich (St. Louis, MO, U.S.A.), and 4′,6-diamidino-2-phenylindole (DAPI) was obtained from Thermo Fisher Scientific.

### Ice Template Preparation and the Fabrication of Scaffolds

Vapor deposition polymerization was performed using a custom-built CVD system equipped with three main zones: a sublimation zone, a pyrolysis furnace, and a deposition chamber. The system was maintained at an operating pressure of approximately 150 mTorr using a vacuum pump system (E2M40, Edwards, U.K.), facilitating the removal of water vapor while maintaining the structural integrity of the ice. Commercial dichloro-[2,2]-paracyclophane was used as a precursor for the deposition of poly*-p-*xylylene (Parylene). The precursor was sublimated at approximately 100 °C and transported via an argon carrier gas controlled by a mass flow controller (MFC; MKS Instruments, USA) at a flow rate of 20 sccm. The vaporized precursor then passed through a pyrolysis zone held at 670 °C, where thermal decomposition produced *p*-xylylene diradicals. The deposition rate was maintained at approximately 0.4 Å/s and monitored using a quartz crystal microbalance (QCM, STM-100/MF, Sycon Instruments, USA).

Ice templates were prepared using several methodological approaches. For the droplet method, solutions and suspensions were sprayed using a commercial sprayer (Kingdom, China) onto hydrophobic poly­(tetrafluoroethylene) (PTFE) sample holders, where the droplets solidified into ice particles with diameters of approximately 500 μm. The cubic template method utilized negative molds of PDMS created following established protocols, with mold dimensions of 400 × 400 × 400 μm^3^. For directional freezing, a temperature gradient was established between designated starting points and end points, with liquid nitrogen introduced near the starting point facilitating directional cooling, resulting in gradual freezing progression.

To prepare the cell-containing scaffolds, ADSCs, DPSCs, PC12 or HUVECs were used at a density of 1–5 × 10^5^ cells per sample. The cells were suspended in cryoprotectant medium consisting of 20.5% w/v dimethyl sulfoxide, 15.5% w/v acetamide, 10% w/v propylene glycol, and 6% w/v PEG in MEM-alpha. For growth factor incorporation, FGF-2 (5 or 10 ng/mL) and Wnt-3a (10 or 60 ng/mL) were dissolved in a buffer solution containing 10% w/v CMC, which was previously loaded into a polydimethylsiloxane (PDMS) mold. The cell suspension was protected using an oil phase system in which the cells were mixed with glyceryl trioleate oil at a ratio of 50 μL of cell suspension to 100 μL of oil. This cell-containing oil mixture was then injected as a droplet into the growth factor solution previously loaded in PDMS molds. The molds were created following previously reported methods with dimensions of 400 × 400 × 400 μm^3^.

For the control group of TCPS, the cell were treat with the same cryoprotectant medium and cultured on the culture plate with the same culture medium corresponded to the differentiation group. All of the TCPS control group cell suspensions were not freeze before culturing, which means the cell suspensions with cryoprotectant medium were directly seed on the culture dish at the same time after the scaffold fabrication. For the control group of TCPS, the cells were treated with the same cryoprotectant medium and cultured on culture dish using the same culture medium as the differentiation group. All cell suspensions of TCPS control group were not frozen prior to culturing. This means that the cell suspensions containing cryoprotectant medium were directly seeded onto the culture dishes at the same time as those of the experimental group. For directional freezing, a temperature gradient was established from one end (starting point) to the other end (end point) by introducing liquid nitrogen near the starting point to facilitate directional cooling.

### Characterizations

Comprehensive material characterization was performed using multiple analytical techniques. The optical microscopy analyses were performed using an Olympus IX83 inverted microscope system (Olympus Corporation, Tokyo, Japan) equipped with an sCMOS digital camera (Hamamatsu ORCA-Flash4.0 V3, 2048 × 2048 pixels, 6.5 μm pixel size). The microscopy setup included a standard light source, such as a halogen lamp, to provide adequate illumination. Various objective lenses, ranging from 10× to 100× magnification, were utilized to examine the samples at differing levels of detail, affording both low and high magnifications as required for particular observations. Scanning electron microscopy (SEM) was conducted using a Nova NanoSEM system (FEI, Hillsboro, OR, U.S.A.) operated at a primary energy of 10 keV and a chamber pressure of 5 × 10^–6^ Torr. The samples were dried overnight in an oven and sputter-coated with platinum or gold prior to imaging. For the cell-containing samples, the samples were fixed with 2.5% formaldehyde and dehydrated using a critical point dryer (K850, Quorum Technologies). Three-dimensional analysis of the interior structure was performed via microcomputed tomography (micro-CT). The samples were scanned using a Bruker SkyScan 1272 system at 2.5 μm pixel resolution, with the voltage set to 40 kVp and the current set to 250 μA for a 10 W output in microfocus mode. Image reconstruction was performed using GPU-Nrecon software (Bruker micro-CT), with ring artifacts and beam-hardening corrections applied. Analysis volumes of 600 × 600 × 600 μm (267 slices) were processed using CTAn 1.18.4 software. Chemical analysis included energy-dispersive X-ray spectroscopy (EDS) performed using the SEM to map elements, including carbon and chlorine from poly*-p-*xylylene, oxygen and sodium from bioglass, and nitrogen and sulfur from proteins/cells. To measure the surface roughness of the prepared coatings, a 3D profile of the area of interest was taken with a VK-9500 laser confocal 3D profile microscope (Keyence, Japan), and the surface roughness and 3D profile images were constructed with a Keyence analyzer (version VK-H1-A9).

### Cell Culture

For the cell culture studies, the base culture medium consisted of MEM-alpha supplemented with 10% FBS and 1% antibiotic-antimycotic mixture. The culture conditions were maintained at 37 °C in an atmosphere of 5% CO_2_ and 95% air with 100% humidity. Specialized media formulations were employed for specific applications. The osteogenic induction medium comprised base medium supplemented with 0.1 × 10^–6^ M dexamethasone, 100 μg/mL l-ascorbic acid, and 10 × 10^–3^ M β-glycerol phosphate. The adipogenic induction medium included base medium supplemented with 10 × 10^–6^ M insulin and 500 × 10^–6^ M 3-isobutyl-1-methylxanthine, along with additional protocol-specific supplements. The neurogenic induction medium included base medium supplemented with 5 × 10^–6^ M all-trans retinoic acid, 20 ng/mL bFGF, and 20 ng/mL EGF. The angiogenesis induction medium included base medium supplemented with 50 ng/mL VEGF, 10 ng/mL bFGF, 1 μg/mL heparin, and 20% FBS. The odontogenic induction medium included base medium supplemented with 0.1 × 10^–6^ M dexamethasone, 100 μg mL^–1^ L ascorbic acid, and 10 × 10^–3^ M β-glycerol phosphate.

For cell coculture, HUVECs and MG-63 cells were prestained with the noncytotoxic dyes CellTracker Green CMFDA and PKH26 Red Fluorescent Cell Linker, according to the manufacturer’s instructions. The cells were then encapsulated in different compartments of the patterned scaffold at initial densities of 1.5 × 10^5^ and 0.5 × 10^5^ cells/cm^2^. Additionally, ADSCs were coencapsulated with HUVECs at a 1:1 ratio to promote and maintain tube network formation on the designated scaffolds. The cocultured samples were incubated with a culture medium composed of an equal volume of MEM-alpha and M199 media supplemented with 10% FBS, 1% antibiotic–antimycotic, 10 mM β-glycerol phosphate, 100 μg/mL l-ascorbic acid, 0.1 μM dexamethasone, 25 U/mL heparin, and 30 μg/mL ECGS for further characterization. To fabricate the compartmentalized scaffold for cell coculture, two distinct cell-contained solution systems were prepared. One solution contained MG-63 cells, and the other contained HUVECs. These cell-containing solutions were independently frozen into predefined shapes to form iced template modules. The modules were then assembled into a modular structure with overall dimensions of 400 × 400 × 400 μm^3^. A central cubic chamber measuring 300 × 300 × 300 μm^3^ was designated for the HUVEC/ADSC compartment, while the surrounding border region was designated for MG-63 cells. This geometric configuration created spatially isolated compartments with naturally formed boundaries. The assembled iced template construct was then subjected to a vapor-phase deposition process, resulting in the formation of a coculture poly*-p-*xylylene scaffold.

Cell viability was assessed using a LIVE/DEAD Viability/Cytotoxicity Kit with visualization by fluorescence microscopy (Leica, Germany). For differentiation, the adipogenic differentiation of ADSCs was evaluated by Oil Red O staining of lipid droplets, with the cell nuclei counterstained with DAPI. Osteogenic differentiation was assessed by immunostaining for OCN, a marker of mature osteoblasts, with cell nuclei counterstained with DAPI. The neurogenic differentiation of PC-12 cells was confirmed by immunostaining for Nestin, a marker of neural progenitor cells, and Tuj-1, a marker of mature neurons, without nuclear counterstaining. Angiogenesis of human umbilical vein endothelial cells (HUVECs) was confirmed by immunostaining for CD31, a marker of endothelial cells, and the cell nuclei were counterstained with DAPI. Odontogenic differentiation of DPSCs was assessed by immunostaining for DSPP and OCN, with the cell nuclei counterstained with DAPI.

All immunofluorescence images of differentiation were captured using a VK-9500 laser confocal microscope with a resolution of 500 dpi. The experimental group comprised scaffold biopsies, which were also captured with a laser confocal microscope at the middle biopsy surface. The control group was cultured in culture dishes, referred to as TCPS controls, and captured with the VK-9500 laser confocal microscope. For quantification, we utilized ImageJ software to compare intensity parameters and transformed the data to a scale of less than 3 orders of magnitude using arbitrary units. This approach minimized errors stemming from contrast variations and ambient light interference. For the measurement of cell length, the length measurement function in ImageJ were employed to compare the practical scale and determine the actual cell length. This function allows for the use of a relative scale to obtain the actual length.

### Statistical Analysis

Statistical analysis was performed using GraphPad Prism (version 7.0) or IBM SPSS Statistics (version 21.0). Unpaired *t* tests were used for two-group comparisons, whereas ANOVA with the least significant difference (LSD) post hoc test was used for multiple group analyses. Statistical significance was set at *p* < 0.05, and the data are presented as the means ± standard deviations from a minimum of three independent experiments unless otherwise specified. Sample size calculations were based on preliminary data when applicable. The described methods represent standardized procedures developed through rigorous optimization and validation processes, ensuring the reproducibility and reliability of the results across multiple experimental iterations. All procedures were performed following institutional guidelines and approved protocols where applicable.

## Data Availability

All the data generated or analyzed during this study are included in this published article and its Supporting Information files.
